# Properties of Non-Aminoglycoside Compounds Used to Stimulate Translational Readthrough of PTC Mutations in Primary Ciliary Dyskinesia

**DOI:** 10.3390/ijms22094923

**Published:** 2021-05-07

**Authors:** Maciej Dabrowski, Zuzanna Bukowy-Bieryllo, Claire L. Jackson, Ewa Zietkiewicz

**Affiliations:** 1 Institute of Human Genetics, Polish Academy of Sciences, 60-479 Poznan, Poland; maciej.dabrowski@igcz.poznan.pl (M.D.); zuzanna.bukowy-bieryllo@igcz.poznan.pl (Z.B.-B.); 2 Primary Ciliary Dyskinesia Centre, NIHR Biomedical Research Centre, University Hospital Southampton NHS Foundation Trust, Southampton SO16 6YD, UK; C.L.Jackson@soton.ac.uk; 3 School of Clinical and Experimental Sciences, University of Southampton Faculty of Medicine, Southampton SO16 6YD, UK

**Keywords:** readthrough, primary ciliary dyskinesia, premature termination codon, aminoglycosides, STOP suppression, rare disease

## Abstract

Primary ciliary dyskinesia (PCD) is a rare disease with autosomal recessive inheritance, caused mostly by bi-allelic gene mutations that impair motile cilia structure and function. Currently, there are no causal treatments for PCD. In many disease models, translational readthrough of premature termination codons (PTC-readthrough) induced by aminoglycosides has been proposed as an effective way of restoring functional protein expression and reducing disease symptoms. However, variable outcomes of pre-clinical trials and toxicity associated with long-term use of aminoglycosides prompt the search for other compounds that might overcome these problems. Because a high proportion of PCD-causing variants are nonsense mutations, readthrough therapies are an attractive option. We tested a group of chemical compounds with known PTC-readthrough potential (ataluren, azithromycin, tylosin, amlexanox, and the experimental compound TC007), collectively referred to as non-aminoglycosides (NAGs). We investigated their PTC-readthrough efficiency in six PTC mutations found in Polish PCD patients, in the context of cell and cilia health, and in comparison to the previously tested aminoglycosides. The NAGs did not compromise the viability of the primary nasal respiratory epithelial cells, and the ciliary beat frequency was retained, similar to what was observed for gentamicin. In HEK293 cells transfected with six PTC-containing inserts, the tested compounds stimulated PTC-readthrough but with lower efficiency than aminoglycosides. The study allowed us to select compounds with minimal negative impact on cell viability and function but still the potential to induce PTC-readthrough.

## 1. Introduction

Motile cilia are specialized, evolutionarily conserved organelles protruding from the apical surface of epithelial cells throughout the human body (the airway, eustachian tubes of the inner ear, female fallopian tubes, brain ependyma, and the embryonic node); the flagella of sperms are structurally the same [1]. Through their coordinated movement, cilia propel mucus, contributing to mucociliary clearance. Primary ciliary dyskinesia (PCD, OMIM#242650) is a rare heterogeneous disease (approximately 1:20,000 live births), predominantly recessive, although X-linked inheritance is known. PCD is caused by mutations in genes that encode essential ciliary structure proteins [2] or proteins involved in cilia assembly [3]. To date, over 50 PCD genes have been reported, mutations in which cause a spectrum of ciliary movement impairments, depending on the ultrastructural defect they cause [4,5]. Clinical symptoms occur from birth (neonatal respiratory distress) and mainly cause recurrent and chronic upper and lower airway infections, which lead to progressive deterioration of pulmonary function if not treated [2]. Disruption of the left–right asymmetry of inner organs (situs inversus) occurs in approximately half of PCD patients, and both male and female fertility can be affected depending on the causal PCD gene [6,7]. A PCD diagnosis is challenging and requires several specialized tests [8]. Only the confirmed presence of bi-allelic (or hemizygous) mutations [9] can prove PCD diagnosis. Patients benefit from an early diagnosis and access to specialist care [10], but currently no causal treatments for PCD exist.

The majority of disease-causing variants are nonsense mutations, which introduce a premature termination codon (PTC) that interrupts proper protein translation [11]. The frequency of particular causative mutations in PCD genes varies highly, depending on the population. Among Polish PCD patients, frequently occurring nonsense mutations are found, e.g., in *SPAG1* (exon 16: p.Gln672ter), *CCDC40* (exon 20: p.Tyr1118ter), *DNAH5* (exon 32: p.Arg1711ter, exon 49: p.Arg2677ter, exon 55: p.Arg3096ter), and *DNAH11* (exon 70: p.Arg3809tyr).

Nonsense mutations are the major cause of cystic fibrosis (CF) or Duchenne’s muscular dystrophy (DMD) [12]; both genes have been targeted for PTC-readthrough therapies to restore functional protein expression and reduce disease symptoms, but pre-clinical trials have shown limited success [13,14]. Chemically stimulated readthrough of in-frame PTCs present in mRNA enables suppression of the PTC by promoting interaction with near-cognate transfer RNAs (nc-tRNAs) and incorporation of an amino acid into the nascent protein chain [15,16]. This allows the ribosome complex to continue translation until the next in-frame STOP codon [17]. This yields a full-length protein, which is often functional, even if the incorporated amino acid differs from the wild type. The method does not affect the genome or the transcriptome of a patient [15,18].

The level of PTC-readthrough depends on many factors, such as PTC type (TAA, TGA, or TAG), its surrounding sequence context, the level of mRNA expression, and the stimulating compound used, as well as other factors, making translational readthrough of each PTC a highly individual case [16].

Aminoglycosides (AGs) such as gentamicin, G418, paromomycin, and amikacin (commonly known antibiotics usually used to treat aerobic Gram-negative bacilli infections) have the ability to stimulate PTC-readthrough [18]. They bind to the A-site of the ribosome [19], inducing a conformational change in the ribosomal decoding center, which facilitates the incorporation of nc-tRNAs and promotes PTC-readthrough [18]. However, AGs are characterized by high nephro- and ototoxicity, which limits their long-term clinical use [20,21].

In our previous work, we analyzed the potential of AGs to stimulate PTC-readthrough of selected PTCs found in several genes causing PCD in the Polish population [22]. While low AG concentrations efficiently stimulated PTC-readthrough in the in vitro system of rabbit reticulocytes, the studies on living cells required much higher, toxic concentrations of AGs, most probably due to their poor capability to penetrate the cell membrane [15]. The results of our study have indicated the need to search for compounds other than AGs that possess lower toxicity and higher potential for PTC-readthrough stimulation [22]. 

In the present study, we tested a group of chemically distinct compounds with proven PTC-readthrough [23,24,25,26] potential, collectively referred to as non-aminoglycosides (NAGs) [27]. We tested the effect of NAGs, compared to that of conventionally used AGs [22], on cell viability and ciliary beating in primary nasal respiratory epithelial cells. The PTC-readthrough-stimulating efficiency of these NAGs was examined in the context of several PTC mutations found in Polish PCD patients.

## 2. Results

### 2.1. Compound Selection

Based on the literature data, we selected several NAG compounds with proven PTC-readthrough-stimulating potential and a reported toxicity lower than that of the AGs. To accelerate the process of clinical approval of the possible therapeutics, we primarily focused on compounds already registered as drugs. We thus selected amlexanox [28,29], a drug with anti-allergic and anti-inflammatory properties; escin, an herbal anti-inflammatory drug [30]; two macrolide antibiotics with PTC-readthrough-inducing properties, tylosin and azithromycin [23,24,31]; and ataluren, the only FDA-registered readthrough-inducing drug (known also as PTC124) [25,32]. Additionally, we used pyranmycin (TC007) [33,34,35], which is an experimental PTC-readthrough-inducing derivative of the AG antibiotic neomycin. The selected concentration range was based on the above literature and cytotoxicity screening with Trypan blue dye assay (Supplementary Table S1). All these compounds have been presented in detail in our review paper [17].

### 2.2. Testing Cytotoxicity of NAGs in Primary Nasal Epithelial Cells from Healthy Donors

**Figure 1:**
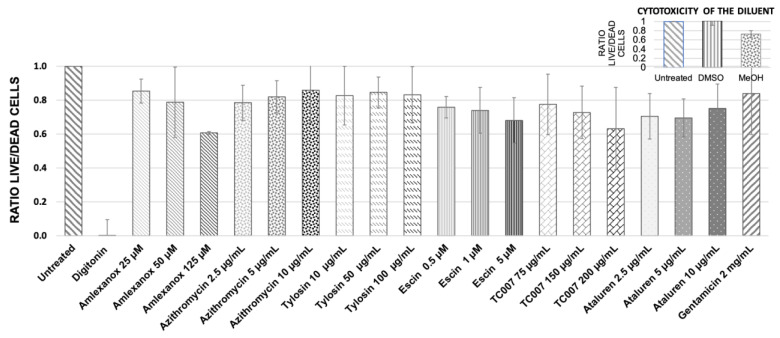
Quantitative estimation of NAG compounds’ influence on the primary RE cells’ viability using Promega Multitox-Fluor cytotoxicity assay. The values shown are the means from 3 independent experiments; error bars show the standard deviation of the mean. The live/dead cell ratio for untreated control equals 1.0; digitonin-treated cells were used as a full cytotoxicity indicator (live/dead ratio = 0.0). The inset represents the influence of diluents on the cell viability.

A Promega Multitox-Fluor cytotoxicity assay was used to simultaneously measure cell viability and death in primary nasal epithelial cells (PNECS) from *n* = 4 healthy donors treated with the following NAGs: amlexanox (125 µM, 50 µM, 25 µM), azithromycin (10 µg/mL, 5 µg/mL, 2.5 µg/mL), escin (5 µM, 2.5 µM, 1.25 µM), tylosin (100 µg/mL, 50 µg/mL, 10 µg/mL), ataluren (10 µg/mL, 5 µg/mL, 2.5 µg/mL), and TC007 (200 µg/mL, 100 µg/mL, 75 µg/mL). Untreated control cells were used as a 100% live reference (with a live/dead cell ratio = 1.0), and cells treated with digitonin 50 µg/mL caused 100% cytotoxicity (live/dead ratio = 0.0). Cells treated with the highest “carrier only” concentration of DMSO (1:280), the diluent for amlexanox, azithromycin, and ataluren, did not exert any cytotoxic effect, as evidenced by a live/dead ratio of 1.0 (Figure 1, inset). The PNECs were, however, susceptible to MeOH (the diluent for escin), which at the highest carrier-only concentration of 1:2000 gave a live/dead ratio of 0.75 (Figure 1, inset). The PNECs were also treated with previously optimized AG (2 mg/mL gentamicin [22]), and the live/dead ratio was 0.86 (Figure 1).

The highest tested concentration of amlexanox was 125 µM (*p* = 0.21); it reduced cell viability to 60%. Therefore, this amlexanox concentration was not analyzed further in the PTC-readthrough tests. A relatively high decrease in the cell’s viability (nearly to 70%) was observed for escin (Figure 1). However, since a similar decrease in viability was also observed for MeOH at the concentration used for escin dilution (Figure 1, inset), we concluded that the observed toxicity of escin in PNECs was primarily associated with the toxicity of the diluent used.

### 2.3. Cilia Motility Analysis

The epithelium lining the airways not only functions as a barrier to prevent the entry of inhaled dust and pathogens into the organism but also actively removes them by the mucociliary clearance mechanism—the coordinated movement of respiratory cilia that propels the mucus along the airways. Even if the tested NAGs induce PTC-readthrough at a therapeutic level, one has to ensure that the potential application of NAGs will not negatively influence the viability of epithelial airway cells (hence cytotoxicity tests above) and that the natural protection mechanisms related to functional airway cilia will be retained (hence cilia motility analysis).

**Figure 2:**
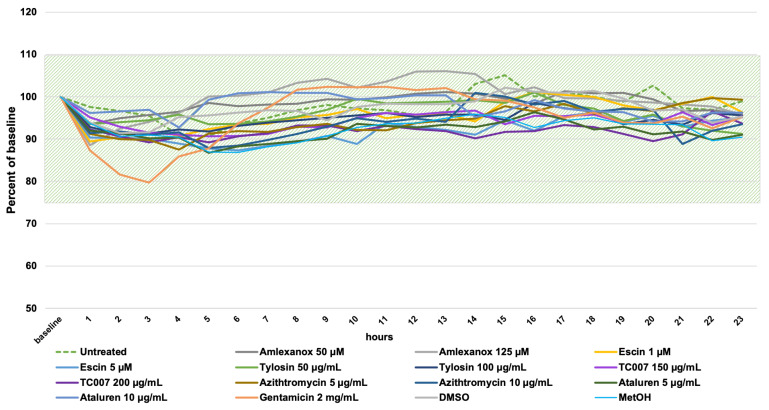
A 24-h time-lapse high-speed video microscopy assessment of ciliary beat frequency (CBF) on PNECs treated with NAGs. The CBF (Hz) measurements were normalized as a percentage of the baseline CBF for each sample, and the single data points are the means of triplicate measurements (of three different x, y, z positions) per sample. The shaded field shows the in-house normal CBF range between 11 and 20 Hz [36]. Please note that the Y axis starts from 50%.

Time-lapse high-speed video microscopy (HSVM) at 37 °C was used to observe and measure the effect of NAGs on the ciliary beat frequency (CBF) as a surrogate for cilia health (with an in-house normal range of 11–20 Hz [36,37]). PNECs were seeded into 24-well culture plates, and using a motorized stage driven with time-lapse software, repeated hourly cilia measurements (at three x, y, z points corresponding to ciliated clusters of PNECs) were recorded for 24 h. Baseline CBFs were recorded prior to PNEC treatment with NAGs or controls. During the observation period, the CBF of cells treated with all amlexanox and gentamicin concentrations did not show significant changes (*p* > 0.05). For all other tested compounds, we observed a slight drop in the CBF from 14.2 Hz ± 0.61 at the baseline to 13.78 ± 0.19 Hz (*p* < 0.001), but this remained within the expected normal CBF range. Variability was observed in the untreated cells (Figure 2, green dotted line); we concluded that the small CBF fluctuations observed after NAG administration were a result of the natural CBF variability. PNECs treated with 2 mg/mL of gentamicin demonstrated a 20% reduction in CBF (13.81 Hz ± 0.85 to 11.04 Hz ± 1.26), which sustained during the first 4 h after compound administration before returning to the baseline CBF (Figure 2, orange line).

### 2.4. PTC-Readthrough Stimulation by NAG Compounds in the HEK293 Cell Line

The PTC-readthrough-stimulating potential of the NAGs was tested in the human epithelial kidney (HEK293) cell line transfected with pDluc vectors. The vectors used in the experiment contained one of six PTC mutations corresponding to those detected in Polish PCD patients (Table 1) or a corresponding wild-type DNA sequence (wt).

The level of PTC-readthrough was assessed by measuring fluc and rluc luminescence in the whole-cell lysates obtained from the NAG-treated HEK293 cultures, as previously described [22]. Fluc reflects the presence of the full-length translation product and rluc that of a short product (translation stopped at the site of PTC). The ratio of fluc to rluc luminescent signals measured in cells transfected with a pDluc vector containing PTC was normalized to the fluc/rluc value in cells transfected with the corresponding wild-type constructs, thus allowing the estimation of the PTC-readthrough efficiency. To compare different PTC-containing vectors and the effectivity of different compounds tested, the efficiency of PTC-readthrough is further displayed as a fold difference between the normalized fluc/rluc ratio in the treated cells versus the untreated cells, both transfected with a specific PTC-containing plasmid.

**Figure 3:**
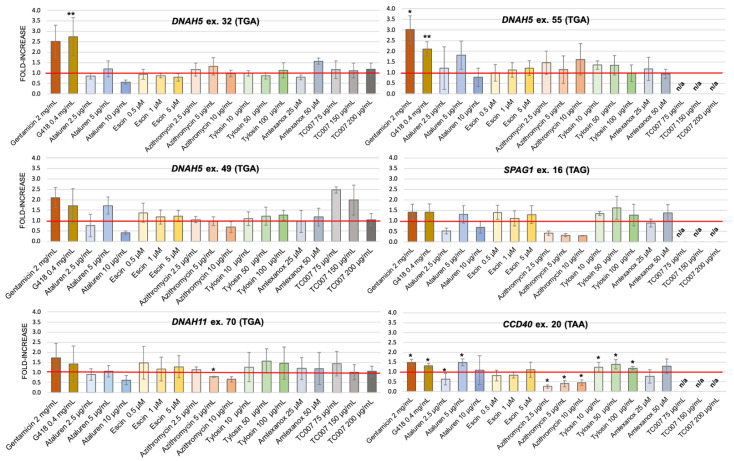
PTC-readthrough stimulation by NAG compounds. The fold increase in PTC-readthrough in treated vs. untreated cells expressing a mutated sequence. HEK293 cells were transfected with a pDluc-PTC construct and incubated with different concentrations of NAGs. n/a—not analyzed. The red line marks samples with PTC-readthrough higher than the basal readthrough (untreated cells transfected with PTC-containing plasmid). * *p* < 0.1, ** *p* < 0.05.

In the majority of cells transfected with PTC-containing plasmids, treatment with NAGs resulted in only a small increase in PTC-readthrough over the values observed in the untreated cells; the size of this stimulating effect was not only small but also depended on the NAG concentration (Figure 3). The level of PTC-readthrough stimulated by any tested concentration of NAGs was generally much lower than for the two AGs used as controls (gentamicin (2 mg/mL) and G418 (4 mg/mL)). The only exception was observed for TC007; its two lower concentrations applied to the cells transfected with plasmids containing the insert with PTC in *DNAH5* ex. 49 resulted in a 2-2.5-fold increase in the PTC-readthrough level (in comparison to a 1.5-2-fold increase in the presence of gentamicin and G418). A 1.5-fold increase in PTC-readthrough was also observed at the lowest TC007 concentration for *DNAH11* ex. 70; no effect was seen in *DNAH5* ex. 32. Unfortunately, three of the remaining PTCs could not be tested with this compound due to the short supply of TC007.

The stimulation efficiency also depended on the PTC identity; the lowest stimulation was observed for PTCs in *CCDC40* ex. 20 (TAA) and *SPAG1* ex. 16 (TAG), regardless of whether AGs or NAGs were used as PTC-readthrough-stimulating compounds. It has to be emphasized that these two PTCs were the only non-TGA stop codons used in the study. Interestingly, for cells transfected with plasmids containing these two PTCs, treatment with any concentration of azithromycin resulted in a strong reduction (>50%) in PTC-readthrough.

## 3. Discussion

The possibility of correcting gene mutations as a potential treatment strategy in PCD has recently gained much interest [2]. However, correction of the mutated PCD genes using lentiviral vectors containing a functional version of the gene [38] or the use of genome-editing techniques, such as transcription activator-like effector endonucleases (TALENs) or CRISPR-Cas9, is not widely applicable because these methods require designing a dedicated construct for each corrected gene and mutation [39,40]. The high heterogeneity of PCD (>50 genes accounting for approximately 70% of well-characterized PCD patients [4]) makes approaches that require targeting specific genes extremely cumbersome. Chemical stimulation of the PTC-readthrough might be a solution because this method is, in theory, gene independent. PTC-readthrough-stimulating compounds should work with any genes if the STOP codon identity and its surrounding nucleotide sequence are favorable [15,16].

The mechanism of action (MOA) of PTC-readthrough is well established for AGs: they bind to the ribosomal A-site and stimulate incorporation of nc-tRNAs due to a conformational change in the ribosomal decoding center [15,16,18]. However, the MOA is elusive for most of the NAGs. For the macrolides, the proposed MOA works either by weakening the binding of the release factor or by disrupting the mechanism of peptide release [41]. The detailed mechanism of action of PTC124 is still debated. Roy et al. proposed that PTC124 binds to the ribosomal A-site and promotes insertion of nc-tRNAs at the STOP codon, similarly to AGs [42]. The other MOA explanation indicates an mRNA as the target for this compound [43]. In this mechanism, the binding of PTC124 to mRNA can lead to mRNA:tRNA mispairing and provoke the insertion of a near-cognate tRNA [44]. For other NAGs, the MOA is still unknown.

In the current study, the effect of NAGs on the efficiency of PTC-readthrough was rather modest, with PTC-readthrough levels mostly below the level achieved by the AGs used as a reference [22], except for compound TC007. Our research showed that of all tested PTCs, only those with the TGA codon were susceptible to the PTC-readthrough. The highest stimulation was observed for PTCs from *DNAH5* ex. 32 and *DNAH5* ex. 49, which contained a TGA codon preceded by purine and followed by cytosine; this phenomenon is well described in the literature [16,45,46,47].

Among all the NAGs tested, TC007 reached or exceeded the PTC-readthrough-stimulating efficiency of the AG compound gentamicin and did not cause adverse cytotoxicity or impair ciliary function in healthy PNEC donor cells. TC007 has also been successful in cellular and murine models of spinal muscular atrophy [26,34]. Of the three different PTCs tested with TC007, high stimulation of PTC-readthrough was observed for PTC in *DNAH5* ex. 49 and for PTC in *DNAH11* ex. 70. Unfortunately, three of the remaining PTCs could not be tested with this compound due to the short supply of TC007.

Surprisingly, a low PTC-readthrough-stimulating efficacy was observed for ataluren, an FDA-approved drug for readthrough therapy in DMD, although this could be a cell-specific effect, since we were assessing use in airway cells (reflecting the proposed site of use). Despite promising results in in vitro experiments and first clinical trials [48,49], recent evidence suggests that the efficiency of PTC-readthrough stimulation by ataluren might be not as high as originally expected. The latest studies on the zebrafish model of DMD have shown that muscle pathology and function of analyzed mutant lines were not improved by ataluren treatment [50]. Clinical studies have also not shown expected results: a phase III clinical study in DMD patients indicated that the effects of ataluren treatment did not differ significantly from the placebo group [51]. Another recent phase III clinical study on CF patients treated with ataluren reported a failure to reach the expected primary and secondary endpoints (ClinicalTrials.gov; id: NCT02139306). In light of these discouraging results, PTC Therapeutics (the manufacturer of ataluren) decided to discontinue its clinical development of ataluren for CF and to withdraw its application for marketing authorization in Europe [52]. A possible explanation of the inconsistency of ataluren studies was proposed by Berger et al. [50]. They suggested that a positive effect of ataluren therapy in some cases might be a result of a completely different mechanism of action, in which alternative translation start points are employed to generate shorter but largely functional dystrophin proteins [50].

Regarding PTC-readthrough efficiency results, a discrepancy was observed between our current study and the previous work focused on the use of AGs as PTC-readthrough-stimulating agents [22]. For example, the highest previously observed stimulation potential, observed for 2 mg/mL of gentamicin and 0.4 mg/mL of G418 resulted in a 2.46- and 5.16-fold increase, respectively, in *DNAH11* ex. 70. In the current study, both compounds yielded only an ~1.5-fold increase, suggesting a much lower stimulating potential of these AGs. The observed discrepancy might be a result of distortions of the fluc/rluc assay; indeed, it has been reported that fluctuation of renilla activities expressed from a pDluc vector may influence the readthrough estimation [53].

An interesting effect was observed for PTCs located in *CCDC40* ex. 20 and *SPAG1* ex. 16, in cells stimulated with azithromycin. The PTC-readthrough stimulation in these HEK293 cell transfectants was much lower than in the untreated cells. Since both PTCs had a STOP codon other than TGA (TAA and TAG, respectively), the observed phenomenon could be caused by ribosome stalling on the STOP codons that are less leaky than TGA. Of note, the antibacterial mode of action of erythromycin, another macrolide antibiotic, is based on this mechanism: erythromycin induces ribosome stalling and translation arrest in the bacterial cells [54]. However, for cells treated with tylosin, the second tested macrolide, no decrease in PTC-readthrough stimulation was observed. Interestingly, when the levels of fluc/rluc were re-examined, we found that the decrease in the level of PTC-readthrough in azithromycin-treated cells transfected with PTC plasmids was caused by the increased fluc/rluc ratio in control cells (transfected with plasmids containing wild-type inserts) rather than by a decreased fluc/rluc ratio in cells transfected with PTC-containing inserts (not shown). An explanation of this aberration could be a post-translational stabilization of the firefly luciferase (fluc), leading to an increase in the fluc activity levels in cells treated with ataluren. Auld et al. observed that ataluren appears to increase, instead of inhibiting, fluc activity in cell-based reporter gene assays and, thus, may distort the results of PTC-readthrough efficiency levels in the assays based on the luminescence of this protein [55].

In bacteria, toxicity of AGs results from a massive suppression of “normal” termination codons (NTC) and deleterious effects of C-terminally extended proteins [15]. However, in eukaryotic cells, AGs’ toxicity appears to be associated with unspecific reactions of AGs with cellular phospholipids, leading to the lysosomal dysfunction or massive generation of reactive oxygen species [17]. AGs might also preferentially bind to the mitochondrial ribosomes of eucaryotic cells due to their similarity to bacterial ribosomes. Such AG binding may first cause protein mistranslation and inhibition of mitochondrial protein synthesis, ultimately leading to cell death [56]. Another known problem with AG-stimulated PTC-readthrough is the fidelity of STOP codon recognition. In eucaryotes, there is a significant difference in the readthrough efficiency of NTCs and PTCs (0.001–0.01% and 0.01–1% of transcripts, respectively), which indicates that premature translation termination differs from the normal termination process [18]. As a result, AG compounds were thought not to significantly affect the misreading of sense STOP codons in eukaryotes [57,58]. However, recent studies by Wangen and Green introduced a plot twist in our understanding of PTC-readthrough [59]. The authors showed that high concentrations of some aminoglycosides (i.e., G418) can induce the readthrough of PTC but also to some extent that of NTC. Most of mRNA transcripts are protected because of the second in-frame STOP codon in 3′UTR, which prevents the translation of mRNAs unnecessarily extended at the C-terminus. However, this does not apply to all transcripts. The PTC-readthrough might perturbate the precise regulation of translation, disturbing several cellular processes and finally inhibiting protein synthesis [59]. Since PTC-readthrough therapy requires the life-long application of stimulating compounds, their specificity of action and low toxicity are essential. Knowing the drawbacks of the therapeutic use of AGs, it becomes important to find a new compound with similar or better PTC-readthrough stimulating potential and reduced negative effects on the cell.

In our previous study, we showed that AGs are able to stimulate PTC-readthrough, but the level of PTC suppression ex vivo was not high enough for therapeutic use [22]. Furthermore, higher concentrations of the tested AGs exerted a toxic effect on epithelial respiratory cells [22]. In the present study, we examined several NAGs with known PTC-readthrough stimulation potential [15,24,25,26,29,43] and analyzed their cytotoxicity and effect on the ciliary beat frequency (indicator of ciliary health), with the hypothesis that they would be less toxic than the AGs typically used for readthrough therapies. Our analysis showed that the cytotoxicity of the tested NAGs (amlexanox, ataluren, TC007, escin, and macrolides azithromycin and tylosin) at the applied concentrations was similar to that of previously used 2 mg/mL of gentamicin [22]. According to the literature, the toxicity of tested NAGs should be significantly lower [15,24,25,26,29]. The maximal toxicity among all NAGs tested was observed for the highest concentration of amlexanox (125 mM), for which reduction to 60% of the viability of primary epithelial cells was observed.

The cytotoxic effect of amlexanox was specific for the renal cell line (HEK293) used for the analysis of the PTC-readthrough potential of NAG compounds. The explanation for this phenomenon might be related to the recent discovery of the anti-tumoral effects of this drug [60], where amlexanox suppressed proliferation and invasion and induced apoptosis in glioblastoma cells. Similar effects could be induced in an immortalized, fast-dividing cell line, such as HEK293. As a result, we excluded the highest concentration of amlexanox (125 µM) from further experiments. This confirms that the toxicity of PTC-readthrough-stimulating compounds remains an important issue related to their potential clinical use and should be assayed on cells from different tissues.

We demonstrated that NAGs exert similar cytotoxic effects as AGs in healthy PNECs, despite the fact that the PNEC viability remains above 65%. According to the literature, an advantage of NAGs compared with AGs is the lack of oto- and nephrotoxicity [15,30], which makes NAGs potentially more appropriate for prolonged therapy requiring repetitive administration. While our finding that TC007 effectively stimulated PTC-readthrough makes this compound an interesting candidate, it has to be kept in mind that TC007 could exhibit ototoxic effects in vivo, similar to its AG precursor antibiotic neomycin [61].

Ciliary integrity can be negatively affected by different chemicals (e.g., flavoring chemicals in electronic cigarettes), air pollutants, and bacterial and viral infections [44,62,63]. Our analysis of the influence of AGs and NAGs on cilia motility showed no significant effect of tested compounds on the CBF during the 24-h testing period. This lack of ciliotoxicity is a promising observation, important for the clinical use of NAGs in PCD patients.

From a long-term perspective, the search for other NAG compounds with higher efficiency in stimulating translational PTC-readthrough of the PCD-causing nonsense mutations should continue, perhaps by looking further into TC007 and acknowledging the possibly nephrotoxic effect of amlexanox. Moreover, studying the influence of NAGs on the viability of the respiratory epithelium and motility of cilia lays a foundation for further work on the therapeutic use of these compounds, both in PCD and in other genetic diseases affecting the respiratory system.

## 4. Materials and Methods

### 4.1. Quantitative Cytotoxicity Analysis

PNECs collected by nasal brushing biopsy from 4 healthy donors were seeded on a 96-well plate in Pneumacult Ex Plus (Stemcell Technologies Inc., Vancouver, BC, Canada) and cultured for 6–9 days to achieve 90% confluence. Then, cells were treated for 48 h with the selected concentrations of various compounds (wells in triplicates). Afterward, the amounts of dead and viable cells were assessed using Multitox Reagent (Promega, Madison, WI, USA) and a Glomax Multi 96-well plate reader (Promega, Madison, WI, USA) according to the manufacturer’s recommendations (viability marker: glycyl-phenylalanylamino fluorocoumarin, GF-AFC, 400Ex/505Em; cell-death marker: bisalanyl-alanyl-phenylalanyl-rhodamine 110, bis-AAF-R110, 485Ex/520Em). Cells treated with 50 µg/mL of digitonin were used as a positive toxicity control and cells treated with 2 mg/mL of gentamicin were used as a reference to compare the effect of NAGs with AGs.

### 4.2. Time-Lapse High-Speed Video Microscopy (HSVM)

Ciliated cells collected by nasal brushings from 3 healthy donors were seeded in a 24-well plate in Basal Epithelial Cell Growth medium (PromoCell, Heidelberg, Germany) [36]. After 24 h, the medium was replaced by Medium 199 (Sigma-Aldrich Corporation, Saint Louis, MO, USA) with different NAG concentrations, and cells were placed in the incubating chamber (37 °C) attached to the stage of the IX81 (Olympus, Shinjuku, Tokyo, Japan) motorized inverted microscope C-mounted to a high-speed video camera PC2 FASTCAM (Photron, Tokyo, Japan). Per well, the localization of three regions of interest (ROIs) containing cell clusters with visible cilia were selected and x, y, z positions were programmed into an automated time-lapse system with a motorized stage (Xcellence rt software, version 2, build 4768, Olympus, Shinjuku, Tokyo, Japan). Ciliary movement at each ROI was recorded for 10 s at 250 frames per second at 1-h intervals for 24 h. To retrieve the ciliary beating frequency (CBF) of the cell clusters, recorded videos were analyzed by automated fast Fourier transform analysis (ImageJ software (https://imagej.net/, accessed on 21 February 2021) with a dedicated CBF Panel plug-in, courtesy of Peter Lackie from the University of Southampton, Southampton, UK) [36]. Individual data points were a mean of three ROIs and experiments were repeated independently with three different PNEC donor samples.

### 4.3. Reporter Vectors Preparation

The construction of reporter vectors has been described in detail in our previous paper [22]. Insert synthesis: 24-nt-long oligonucleotides representing PTC-containing and wild-type DNA sequences of the analyzed PCD genes were chemically synthesized (Genomed, Warszawa, Poland). Oligonucleotides for both forward and reverse strands were prepared, with 4 bp overhangs (upper strand: 5′TCGAG; lower strand: 5′GATCT) added to enable ligation into the digested plasmid. Insert cloning: phosphorylated and duplexed oligonucleotides were ligated into the XhoI- and BglII-digested polylinker region of pDluc, a dual-luciferase reporter vector (kind gift of Prof. John Atkins from the University of Utah). After ligation, 100 ng of each construct was electroporated (2.5 kV, 25 µF, 200 Ω) into the *Escherichia coli* strain DH5α. Plasmids were amplified in *E. coli* and purified using a Midiprep Kit (Qiagen, Hilden, Germany) according to the manufacturer’s recommendations. All insert sequences were confirmed by direct dideoxy sequencing.

HEK293 cells were cultured in DMEM (Sigma-Aldrich Corporation, Saint Louis, MO, USA) supplemented with 10% fetal calf serum (FCS), 1% GlutaMAX, and MEM non-essential amino acids (all three from ThermoFisher Scientific, Waltham, MA, USA), and 1% Pen-Strep (Sigma-Aldrich Corporation, Saint Louis, MO, USA). The cells were grown at 37 °C in a humidified atmosphere containing 5% CO_2_; the medium was changed three times a week.

A day before transfection, the cells were suspended in the culture medium at a concentration of 10 × 10^6^ cells/mL and seeded into a 24-well plate at a density of 22 × 10^3^ cells per well. The cells were transfected with the mixture of a specific vector and the JetPrime transfection reagent (Polyplus-Transfection, Lllkirch, France) (0.25 ng DNA/well; DNA/JetPrime ratio 1:4) 24 h later.

### 4.4. PTC-Readthrough-Stimulating Potential Measurement

One day after transfection, fresh growth medium, with or without NAGs (Table 1), was added to the cells and incubated for the next 24 h. Each combination of construct–NAG was assayed in three independent wells of the plate. After 24 h of incubation with NAGs, cells were lysed using 100 µL of 1× Passive Lysis Buffer (Promega, Madison, WI, USA) and the PTC-readthrough efficiency was measured using the dual-luciferase reporter assay and the dedicated Glomax Multi Detection System with injectors (Promega, Madison, WI, USA). Each lysate was measured in triplicate, using 20 µL of the lysate and 50 µL of dual-luciferase reporter assay reagents at the Glomax acquisition settings recommended by the manufacturer.

To estimate the efficiency of PTC-readthrough stimulation, the ratio of fluc activity to rluc activity in the lysates of NAG-treated cells transfected with a PTC-containing construct was calculated. Then, the values were normalized to the fluc/rluc ratios obtained from the cells transfected with the corresponding wild-type construct (not treated or treated with NAG). The PTC-readthrough efficiency was shown as a fold-increase over untreated cells. All reported values were the average of at least three experiments. TC007 was tested only for three PTCs (*DNAH5* ex. 32 and 49 and *DNAH11* ex. 70) because of the limited stock of this experimental compound.

### 4.5. Statistical Analysis

All statistical analysis were performed using Statistica software v.13.3 (TIBCO Software Inc., Palo Alto, CA, USA). Several statistical tests were used during the study: Kruskal–Wallis test for analysis of cytotoxicity, Kolmogorov–Smirnov test analysis of readthrough efficiency statistical significance, and Student’s *t*-test for time-lapse HSVM analysis. Asterisks in figures denote significance levels of * *p* < 0.1 and ** *p* < 0.05.

## Figures and Tables

**Table 1:** PTC mutations tested corresponding to those detected in Polish PCD patients.

Gene/Exon	refSNP (If Available)	Nucleotide Substitution	AA Change	nt −3	nt −2	nt −1	PTC	nt +4	nt +5	nt +6
*DNAH5* ex. 32	-	c.5131C/T	Arg1711ter	A	A	A	TGA	C	T	G
*DNAH5* ex. 49	rs775946081	c.8029C/T	Arg2677ter	G	T	G	TGA	C	A	G
*DNAH5* ex. 55	-	c.9286C/T	Arg3096ter	T	T	T	TGA	A	A	C
*DNAH11* ex. 70	-	c.11425C/T	Arg3809ter	C	T	T	TGA	T	T	C
*SPAG1* ex. 16	rs201740530	c.2014C/T	Gln672ter	T	G	C	TAG	T	T	T
*CCDC40* ex. 20	rs374909386	c.3354C/T	Tyr1118ter	G	A	G	TAA	C	C	C

Each PTC is named by gene and exon. The neighboring nucleotide sequence is numbered with respect to the PTC localization, nucleotide 1 being the first nt of the STOP codon. AA—amino acid; refSNP—reference number in the SNP database.

## Data Availability

Data supporting reported results are available upon request.

## Supplementary Materials

The following are available online at https://www.mdpi.com/article/10.3390/ijms22094923/s1, Table S1: Exclusion of NAG concentrations that caused less than 50% 16HBE14o- cell survival.

## Abbreviations

PCD	primary ciliary dyskinesia
PTC	premature termination codon
AG	aminoglycoside
NAG	non-aminoglycoside
DMD	Duchenne’s muscular dystrophy
CF	cystic fibrosis
PNEC	primary nasal epithelial cells
MeOH	methanol
HSVM	high-speed video microscopy
CBF	ciliary beating frequency
MOA	mechanism of action
NTC	normal termination codon
