# Supplementary material for: Properties of Non-Aminoglycoside Compounds Used to Stimulate Translational Readthrough of PTC Mutations in Primary Ciliary Dyskinesia
Source: Int J Mol Sci. 2021 May 7;22(9):4923. doi: 10.3390/ijms22094923 (PMC8125088; doi:10.3390/ijms22094923)
Supplement: Supplementary file 1 [file ijms-22-04923-s001.zip › ijms-1171717-supplementary.pdf]

Pre-screening cytotoxicity of NAGs in the 16HBE140- cell line

Based on the available literature data, five initial concentrations of each compound were tested on the human bronchial epithelial 16HBE14o- cell line. Using a Trypan Blue cell exclusion dye assay, to indicate cytotoxicity (blue cells are compromised), we screened NAGs at a dose range previously reported [24,25,26,29,30,35] and found the three highest concentrations that permitted survival of >50% (arbitrarily judged by eye) of the 16HBE14o- cells in submerged culture (Supp. table 1).

Supp. table S1. Exclusion of NAG concentrations that caused less than 50% 16HBE14o- cell survival.

**Amlexanox (µM)** in DMSO	***500***	***250***	125	50	25
**Azithromycin (µg/ml)** in DMSO	***50***	***25***	10	5	2.5
**Escin (µM)** in MeOH	***20***	***10***	5	2.5	1.25
**Tylosin (µg/mL)** in water	***200***	100	50	10	N/A
**Ataluren (µg/mL)** in DMSO	***20***	10	5	2.5	N/A
**TC007 (µg/mL)** in water	200	100	75	N/A	N/A

NAG concentrations that caused less than 50% cell survival are shaded in grey. Diluents (dimethylsulfoxide (DMSO), methanol (MeOH), water) did not exceed 0.35% vol/vol. N/A – not analyzed.
